# The effects of Psychotropic drugs On Developing brain (ePOD) study: methods and design

**DOI:** 10.1186/1471-244X-14-48

**Published:** 2014-02-19

**Authors:** Marco A Bottelier, Marieke LJ Schouw, Anne Klomp, Hyke GH Tamminga, Anouk GM Schrantee, Cheima Bouziane, Michiel B de Ruiter, Frits Boer, Henricus G Ruhé, Damiaan Denys, Roselyne Rijsman, Ramon JL Lindauer, Hans B Reitsma, Hilde M Geurts, Liesbeth Reneman

**Affiliations:** 1 Department of child- and adolescent psychiatry, Triversum, Alkmaar, the Netherlands; 2 Brain Imaging Center, Academic Medical Center, University of Amsterdam, Amsterdam, the Netherlands; 3 Department of Radiology, Academic Medical Center, University of Amsterdam, Amsterdam, the Netherlands; 4 dArc (Dutch Autism and ADHD research center), University of Amsterdam, Amsterdam, the Netherlands; 5 Department of Child and Adolescent Psychiatry, Academic Medical Center, de Bascule, Amsterdam, the Netherlands; 6 Department of Psychiatry, Academic Medical Center, Amsterdam, the Netherlands; 7 Center for Sleep and Wake disorders, Medical Center Haaglanden, the Hague, The Netherlands; 8 Julius Center for Health Sciences and Primary Care, University Medical Center Utrecht, Utrecht, the Netherlands; 9 Faculty of Social and Behavioral studies, Brain and Cognition, Department of Psychology, University of Amsterdam, Amsterdam, the Netherlands; 10 Netherlands Institute for Neuroscience, an Institute of the Royal Netherlands Academy of Arts and Sciences, Amsterdam, the Netherlands; 11 Department of Psychiatry, University of Groningen, University Medical Center Groningen, Mood and Anxiety Disorders, Groningen, the Netherlands

**Keywords:** Development, Antidepressants, Psychostimulants

## Abstract

**Background:**

Animal studies have shown that methylphenidate (MPH) and fluoxetine (FLX) have different effects on dopaminergic and serotonergic system in the developing brain compared to the developed brain. The effects of Psychotropic drugs On the Developing brain (ePOD) study is a combination of different approaches to determine whether there are related findings in humans.

**Methods/Design:**

Animal studies were carried out to investigate age-related effects of psychotropic drugs and to validate new neuroimaging techniques. In addition, we set up two double-blind placebo controlled clinical trials with MPH in 50 boys (10–12 years) and 50 young men (23–40 years) suffering from ADHD (ePOD-MPH) and with FLX in 40 girls (12–14 years) and 40 young women (23–40 years) suffering from depression and anxiety disorders (ePOD-SSRI). Trial registration numbers are: Nederlands Trial Register NTR3103 and NTR2111. A cross-sectional cohort study on age-related effects of these psychotropic medications in patients who have been treated previously with MPH or FLX (ePOD-Pharmo) is also ongoing. The effects of psychotropic drugs on the developing brain are studied using neuroimaging techniques together with neuropsychological and psychiatric assessments of cognition, behavior and emotion. All assessments take place before, during (only in case of MPH) and after chronic treatment.

**Discussion:**

The combined results of these approaches will provide new insight into the modulating effect of MPH and FLX on brain development.

## Background

The brain in development is dependent on the emergence of critical developmental processes (i.e. synaptogenesis, [1], and therefore sensitive to pharmacological interventions. Treating children and adolescents with serotonergic (5-HTergic) or dopaminergic (DAergic) drugs like fluoxetine (FLX) and methylphenidate (MPH), is therefore likely to have influence on the maturation of the brain.

For the 5-HTergic system, FLX (a selective serotonin reuptake inhibitor (SSRI), registered for the treatment of depression in children aged 8 years and older, is known to increase extracellular levels of 5-HT by blocking the serotonin transporter (SERT). However, animal studies have demonstrated that periadolescent 5-HT pharmacological manipulations can lead to abnormal outgrowth of the 5-HT system [2,3]. Experiments by our group have shown that chronic treatment with FLX results in a significant increase in prefrontal and hypothalamic 5-HT transporter (SERT; +30%, p < 0.01) in juvenile-treated rats, but not in adult treated rats [4]. These findings are in accordance with Wegerer and Bock who have also shown that this effect persists into adulthood, long after discontinuation of treatment with SSRIs [5,6]. Recently it was confirmed that FLX administration upregulates SERT long-lastingly, also in non-human primates [7]. These preclinical studies suggest that 5-HT manipulations have an impact on the regulation of 5-HT outgrowth which is dependent on the age of exposure.

For the DAergic system, recent animal studies with MPH, a DA reuptake inhibitor and stimulant drug frequently prescribed in the treatment of attention deficit hyperactivity disorder (ADHD), have demonstrated that these effects are also age-dependent. For instance, early treatment with MPH led to a considerable (-50%) reduction of dopamine transport density (DAT) in rat striatum when compared to non-treated animals, whereas no effects were observed in adult animals [8]. These alterations in the DA system have been shown to result in behavioral abnormalities. For example, young rats treated with MPH show more anxiety- and depression-related behavior in adulthood than adult rats treated with MPH [9].

There is some clinical evidence for related findings in humans. For example, after concerns about increased suicide risk among children and adolescents treated with SSRIs, the Food and Drug Administration and European Medicines Agency (EMEA) stated in 2003–2004 that SSRIs were contraindicated for treating depression in children and adolescents. Furthermore, in the NIMH Collaborative Multisite Multimodal Treatment Study of Children With Attention-Deficit/Hyperactivity Disorder (MTA) children who received behavioral therapy had a lower rate of diagnoses of anxiety or depression (4.3%) than the children who were treated with MPH (19.1%) thus indicating a (transient) increase in the occurrence of emotional disorders six to eight years after treatment with MPH [10]. Age-related differences have also been found between adolescent and adult patients on fMRI studies, with adolescent patients treated with MPH showing more activity in the prefrontal cortex after treatment than adult patients [11].

Thus, evidence is slowly emerging that the long-term effects of drug exposure are delayed and come to expression once the vulnerable system reaches maturation (i.e., typically during adulthood). This phenomenon is known as ‘neuronal imprinting’ and occurs when the effects of drug exposure outlast the drug itself [12]. Still, very little is known on exposure during later brain development. Most (clinical) studies are hampered by the fact that they are retrospective in design, and therefore the findings could be caused by other factors on which the groups differed. As pointed out by Shaw and colleagues: ‘….the ideal study design for this question would be a randomized trial comparing cortical growth in children on psychostimulants against an unmedicated comparison group—but this would be both logistically and ethically challenging’ [13]. Notwithstanding this challenge, we have set up three studies (the effects of Psychotropic drugs On the Developing brain ‘ePOD’ project): two randomized controlled trials (RCTs) and a retrospective cohort study, investigating the possibility of the existence of neuronal imprinting in children medicated with these drugs while using several modalities to assess neurocognitive development. Here we report on the objectives and methods of these studies.

### Objectives

#### ***Primary objectives***

1. The primary objective of the ePOD studies is to report on the short-term age-dependency of the effect(s) of MPH treatment on the developing DA system and on the age-dependency of the effect(s) of FLX on the developing 5-HTergic system, using pharmacological MRI (phMRI) as our main outcome measure.

2. Furthermore, we aim to study the long-term effects of these drugs in a cohort study based on medical prescription data.

#### ***Secondary objectives***

Our secondary objectives are:

1. To report on the age-dependency of MPH and FLX on the outgrowth of the DA system and the 5-HT system using functional outcome measures (diffusion tensor imaging [DTI], functional MRI [fMRI], restingstate-fMRI [rs-fMRI] and neuropsychological assessment (NPA)).

2. To report on the age-dependency of the effects of FLX on 5-HT driven HPA axis activity using cortisol measures.

3. To report on the role of the 5-HTTLPR polymorphism upon the age-dependency of FLX on the outgrowth of the 5-HT-ergic system.

4. To report on the effects of MPH on restless legs (RLS) symptoms and insomnia.

## Methods/Design

### General design of the ePOD project

Only a long-term prospective study in patients randomly assigned to MPH or SSRIs and placebo conditions can determine unequivocally whether the (adverse) effects of these medications on the neurotransmitter systems interact with the age when these drugs are prescribed. To this purpose we designed two RCTs, one with MPH and one with FLX. However, it would not be ethical to deprive subjects in a placebo setting from treatment for extensive periods of time. Therefore, in addition to the RCTs, which will last 4 months, we investigate the long-term effects (at least 7 years) in a cohort study based on medical prescription (the ePOD-Pharmo study). The three sub-studies of the ePOD project include:

• ePOD-MPH: A 16 week RCT with MPH in 100 medication naïve ADHD patients. This RCT involves three separate NPA and MRI assessments: the first before starting with the study medication (baseline session), the second during treatment with MPH or placebo (week 8) and the final assessment after trial end following a 1-week washout period (week 17).

• ePOD-SSRI: A 16 week RCT with FLX in 80 medication naïve patients suffering from MDD and anxiety disorders (AD). It involves two seperate NPA and MRI assessments: before starting with the study medication (baseline session) and after treatment with FLX following a 3-week washout period (week 19).

• ePOD-Pharmo: A cohort study based on medical prescription data. One hundred and fifty subjects will be recruited through a database containing prescription data on MPH or FLX (and other antidepressants) Subjects in this cohort based study will receive the same assessments as in the RCTs but only once.

#### ***Randomized controlled trials: design and study samples***

**Figure 1:**
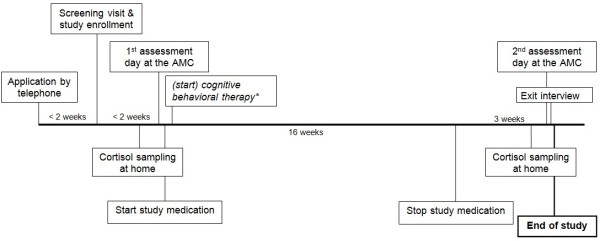
Timeline study procedures SSRI trial; *only in adolescents.

The two RCTs consist of 16-week multicenter randomized, double blind, placebo-controlled trials with a washout period of one week (MPH) or three weeks (FLX). Subjects are stratified into two age categories: MPH: boys aged 10–12 years, and adults aged 23–40 years. FLX: girls aged 12–14 years and adults aged 23–40 years. These two age groups are randomly assigned to either placebo or active treatment. MRI and NPA assessments will take place before treatment (baseline), during treatment (only in the MPH trial) and following the washout period (see Figure 1 for the timeline for ePOD-SSRI RCT). Baseline measurements will be compared with the results obtained at trial end, and for the ePOD-MPH RCT also during the trial. Differences in outcome measures will be compared between the two age categories (children vs. adult), in addition to healthy controls (separate study). In view of our hypothesis that the active treatment results in long lasting or even permanent changes in the developing brain, we expect no or a small change in change scores between baseline-, and post-treatment assessments, whereas in children we expect to find larger changes, as enduring changes will have taken place in the developing brain, but only transient accommodation in the developed brain. Washout periods were chosen based on chemical properties (rate of elimination based on five half–live times) and ethical considerations (time without treatment).

A total of 50 children (10–12 years of age) and 50 adult (23–40 years of age) male outpatients diagnosed with ADHD (all subtypes) and in need of pharmacological therapy will be included in ePOD-MPH RCT. A total of 40 adolescent (12–14 years of age) and 40 adult (23–40 years of age) female outpatients with moderate to severe MDD or an anxiety disorder in need of pharmacological treatment will be included in ePOD-SSRI RCT. Patients that have used medications or drugs that influence the monoamine systems before age 23 are not eligible.

Patients are recruited from clinical programs at the Child and Adolescent Psychiatry Center Triversum (Alkmaar), from the department of (Child and Adolescent) Psychiatry of the Bascule/AMC (Amsterdam), and from PsyQ mental health facility in The Hague. The diagnosis is made by an experienced psychiatrist based on the Diagnostic and Statistical Manual of Mental Disorders, (DSM-IV), Fourth Edition, [14] and confirmed by a structured interview: Diagnostic Interview Schedule for Children (National Institute of Mental Health Diagnostic Interview Schedule for Children Version IV (NIMH-DISC-IV, authorized Dutch Translation) [15], in children or in parents and the Diagnostic Interview for Adult ADHD (DIVA) [16] in adults in the RCT with MPH. For the ePOD-SSRI trial we use the Diagnostic Interview Schedule for Children in children and in adults the Composite International Diagnostic Interview (CIDI; lifetime version 2.1 authorized Dutch translation) [17]. In addition, children must have a Children’s Depression Rating scale-Revised (CDRS-R) [18] score of > 45, and a Children’s Global Assessment Scale (CGAS) [19] score < 50. In adults, a Hamilton Rating Scale for Depression (HRSD-17) [20] ≥18, a Clinical Global Impression scale (CGI) [21] > 4, and or Hamilton anxiety scale (HAM-A) > 20 [22] are required for study inclusion. Subjects must exhibit stable dysphoria/depressed mood and/or anhedonia for at least 2 weeks prior to enrollment and mood should be pervasive (defined as present most of the time in at least two of three contexts: at home, at school or with friends). In both RCTS, patients with co-morbid axis I psychiatric disorders requiring treatment with medication at study entry, with IQ lower than 80 (as measured by a subtest of the Wechsler Intelligence Scale for children-Revised (WISC-R), National Adult Reading Test (NART), authorized Dutch translation [23] and MDD patients with current risk of suicide attempt are excluded.

We chose to include only male patients in ePOD-MPH to limit subject variation and because ADHD is most prevalent in males [24]. Thus, to keep our sample as homogenously as possible and prevent inclusion problems, only male subjects are included in the ePOD-MPH study. The cut-off point of 10–12 years of age was chosen because peak prevalence of ADHD is 10 years of age [25] and also because several MRI parameters greatly change until 8–10 years of age [26], whereas the rate of increase of neuronal growth and pruning reduces after 10 years of age. The cut off point of 23 years for matured brain in the adults is chosen in line with previous studies involving a comparison between matured versus immature brain [27].

Only female subjects are included in ePOD-SSRI based on the higher prevalence of MDD and AD in this population [28]. Thus, to keep our sample as homogenously as possible and prevent inclusion problems, only female subjects are included in the ePOD-SSRI study. For the adolescent group we chose a cut-off point of 12–14 years of age because the risk of MDD and AD onset increases approximately 8 fold at this age compared to children younger than 10 years of age [29,30].

#### ***Cohort based study: design and study sample***

In the ePOD-Pharmo study, we investigate the long-term effects of age following SSRI or MPH treatment on our main outcome parameter (phMRI). Exposed subjects are stratified into two age groups: one group that has been prescribed early in life with these medications, and another group late in life. Subjects are recruited through a medical prescription database from the Pharmo Institute (Utrecht, the Netherlands). This out-patient pharmacy database is a database that contains drug dispensing data since 1986 from over 3 million residents in the Netherlands, corresponding to approximately 20% of the Dutch population. The dispensing date, prescriber, prescribed dosage regimen, and duration are known. Subjects participate in a single assessment day (cross-sectional design) with similar NPA and MRI investigations as in the ePOD RCTs, mentioned above. Subjects eligible for study participation are 23–40 years of age and presumably diagnosed with ADHD or MDD/anxiety disorder. The early exposed group contains subjects with a history of MPH (male subjects) or SSRI treatment (female subjects) before the age of 16 (thus at least 7 years ago). The late exposed group contains subjects treated between 23 and 40 years of age. The early-, and late exposed groups will be compared to an age-, and gender matched unexposed control group, consisting of medication naive subjects suffering from ADHD or MDD/anxiety disorder. Every group (six in total) will contain 25 subjects.

### Assessments

#### ***Clinical rating scales***

For both RCTs we use a set of clinical rating scales to assess symptom severity and functioning at baseline and after treatment. For each study separately we have some additional, disorder-specific rating scales. In the ePOD-MPH RCT, an authorized Dutch translation of the Disruptive Behavior Disorders Rating Scale (DBD-RS) [31] will be used in children and in adults the ADHD-SR [32]. Clinical improvement will be rated in both RCTs by the clinician using CGAS [19] and CGI [21] scales in children, and in adults using the Global Assessment of Function [33]. In both RCTs, the Children’s Depression Inventory (CDI) [34] and the Screen for Child Anxiety Related Emotional Disorders (SCARED) [35] will be administered to children, and the Beck Depression Inventory (BDI) [36] and the Beck Anxiety Index (BAI) [37] to adults. These rating scales are also administered in the ePOD-Pharmo study.

#### ***Imaging parameters***

Imaging parameters are directed towards the DAergic and 5-HTergic system. DAergic and 5-HTergic brain activity will be assessed using phMRI, which is the primary outcome measure of the ePOD project. In addition, DA connectivity will be assessed using rs-fMRI and DTI, and functional brain activity using DA-related (motor inhibition) or 5-HT-related (emotional processing) fMRI tasks. Due to time restrictions of the ePOD-SSRI scan protocol, 5-HT connectivity will only be assessed using DTI.

#### ***phMRI***

Application of fMRI in combination with a pharmacological challenge (phMRI) has the potential to provide an index of changes in neurotransmitter function. With phMRI a neurotransmitter specific pharmacological challenge is given, which causes changes in neurovascular coupling and subsequent region-specific changes in brain hemodynamics. It differs from fMRI, in that the neuronal system is not activated by a motor or cognitive task, but pharmacologically. phMRI has been shown to adequately assess the DA integrity and functionality, as DA-lesioned primates showed a blunted hemodynamic response to a d-amphetamine challenge, following DA lesioning, which correlated strongly with DA transporter availability and motor function [38]. During the phMRI scan, after several minutes of baseline scanning, subjects will receive an oral dose of MPH (0.5 mg/kg with a maximum dose of 20 mg in children and 40 mg in adults). This challenge dose was chosen as it induces maximum blockade of the DAT (80% occupancy), which occurs at serum concentrations of about 8–10 ng/ml. Higher concentrations are not likely to be very effective in further blocking DAT [39]. After 90 minutes, subjects will undergo a second MRI session, and the same MRI sequences are repeated, now under the influence of MPH. The 90 minute time window was chosen, because DAT occupancy is significantly correlated with plasma concentration of MPH, which peaks between 1 and 2 hours following ingestion of MPH [40,41]. DAT occupancy has also been shown to be relatively stable between 1 and 2 hours after ingestion of MPH [40]. Based on the literature (reduction in DAT densities in young, but not adult treated animals) [42] and experiments from our own group in d-amphetamine users with phMRI and a MPH challenge [43], we expect that treatment with MPH will induce a long-lasting changes in the brain hemodynamic phMRI response in DA rich brain areas (e.g. striatum) in children, but not adults. We expect that in adult patients MPH will be accommodated by a series of transient compensatory reactions. However, in children MPH will induce changes in the form of long-lasting developmental alterations of the system, reflecting existence of ‘neuronal imprinting’ in the human brain [44].

In the ePOD-SSRI study, an intravenous (i.v.) challenge with citalopram (5 mg in adolescents and 7.5 mg in adults) will be administered during a single scanning session. A 5-HT challenge is subject to more variability and therefore needs to enter the brain in a rapid and consistent manner over the time course of a single scan session, which requires intravenous administration [45]. Citalopram is currently the only SSRI registered for i.v. administration. When used for therapeutic purposes, intravenous citalopram is given at the same dose as the oral route of administration and it is well within the therapeutic range even for children [46]. Citalopram increases 5-HT release by inhibiting the reuptake of 5-HT by SERT. It has been used previously in phMRI studies and has been proven an adequate probe of 5-HT function [45,47]. We have previously shown that phMRI is able to detect 5-HT neuronal imprinting effects: in young rats chronic FLX treatment resulted in an increased 5-HT reactivity as measured with phMRI, whereas in adult animals FLX it reduced 5-HT brain activity [48]. We expect an increased signal in 5-HT rich brain areas (e.g. prefrontal cortex, hippocampus and hypothalamus) after 5-HT challenge only in FLX treated adolescents when compared to pretreatment baseline scans, in line with our previous findings in rats [48].

#### ***rs-fMRI***

A relatively new fMRI approach (i.e., resting-state fMRI (rs-fMRI)) allows assessment of changes in organization of whole functional networks, including DAergic and 5-HTergic networks. Rs-fMRI detects baseline brain activity related to ongoing neuronal signaling at “rest” and is performed by low-pass filtering of spontaneous blood oxygenation level-dependent (BOLD) fMRI signals [49]. A decreased functional connectivity between anterior cingulated cortex and precuneus has been found using this technique in adult ADHD patients [50]. There are a number of studies that have investigated the effects of MPH or SSRIs on this parameter [14,51-55], which found that these drugs normalize brain activation and functional connectivity abnormalities in patients suffering from ADHD or MDD. In accordance with this literature, we expect to find age-dependent normalization of functional connectivity abnormalities.

#### ***DTI***

With diffusion tensor imaging (DTI), the micro-structural organization of white matter (WM) can be visualized. By measuring the diffusion motion of water molecules, and the fact that this motion is restricted by myelin sheaths, an impression of axonal direction and integrity can be obtained [56]. Fractional anisotropy (FA) is the most commonly used readout marker in DTI and provides information about the degree of fiber organization and integrity. Any process that results in alterations in axonal architecture, such as decreased axonal outgrowth, can result in decrease in FA [57-59]. A previous DTI study in children suffering from ADHD, observed an increase, or rather normalization, of white matter volume in ADHD medicated children compared to unmedicated children [60]. In line with this, chronic treatment with MPH in pre-adolescent rats was found to increase (fold change >1.5) genes involved in striatal growth of novel axons [61]. Furthermore, in a recent study in rats we observed opposite effects of MPH on FA measures: MPH induced an increase in FA in the corpus callosum of adolescent rats, whereas a slight reduction in adult animals [62]. Therefore, we also expect to find age-related findings in the current RCT with MPH: an increase in FA in MPH treated children when compared to pre-treatment baseline scans, and no effect or a small effect in adult patients.

Considering the 5-HTergic system, we have previously shown that alterations in axonal integrity linked to the 5-HTergic system can be adequately assessed using DTI [59]. We hypothesize that chronic treatment with SSRIs leads to increased outgrowth of the 5-HT system, since 5-HT acts as a growth factor in the maturing brain [63]. Therefore we expect an increase in FA (reflecting 5-HT neuronal growth) in 5-HT rich brain areas only in FLX treated adolescents when compared to pretreatment baseline scans. Like for MPH, no effect of treatment on these scan parameters are expected in adults.

#### ***fMRI***

We have selected two task-related fMRI scans either based upon their involvement of the DA system and/or the 5-HT system and the known interaction with MPH or FLX and treatment response in anxiety and depressive disorders. In view of our hypothesis we expect to find a normalized pattern of activation on these tasks in children during treatment, which will persist after the end of the trial. In contrast, the activation pattern in adult subjects will normalize during the trial and fall back to pre-treatment (hypoactivation) values after the end of the trial. The fMRI tasks consist of the following:

An emotional processing task (MPH and SSRI trials, and ePOD-Pharmo study): The BOLD response to negative emotional faces (angry and fearful faces) is measured in a block-design fMRI task [64]. Emotional responses are elicited in many different brain regions, where the amygdala seems to be a relay between visual systems en modulatory responses. Emotional processing is known to be regulated by 5-HT and to be affected in mood disorder [65]. MDD patients are believed to express a heightened responsiveness to negative emotional stimuli and a reduced detection of positive affect [66,67], which is explained by hyperactivity of the affective neurocircuitry, including the amygdala [68]. SSRIs have been found to decrease amygdala activity in response to negative affect in both healthy subjects and MDD patients, which might (partially) explain symptom remission following antidepressant treatment in MDD [69-71].

A motor inhibition task (only MPH trial and ePOD-Pharmo study): Frontal–striatal function and its modulation by MPH will be assessed using a motor inhibition task: the go/no-go task [72]. MPH has been shown to normalize striatal hypoactivation in ADHD subjects [73]. Specifically, fronto–striatal activation during response inhibition will be measured on two versions of a go/no-go task, each with and without administration of MPH. The effects of MPH on frontal and striatal activation during response inhibition will be compared within and between groups.

#### ***Neuropsychological tests***

A neuropsychological test battery (Standard Reaction Time Task, Rey Auditory Verbal Learning Task [74], Sustained Attention to Response Task (SART) [75], N-back (working memory task) [76], Maudsley Index of Delay Aversion (MIDA) [77]) will be administered, addressing reaction time, verbal memory, sustained attention, working memory and delay aversion in particular. This information can be linked to results from imaging in order to determine any links between behavioral and fMRI data and changes in the monoamine systems. We will look for correlations between altered cognitive responses and fMRI responses, phMRI responses, DTI measures and rs-fMRI response.

#### ***Actigraphy and sleep log***

Restless Legs Syndrome is a chronic progressive neurological disorder that has a greater incidence in ADHD children, adolescents and adults than in the general population [78]. It is possible that RLS is co-morbid with ADHD or that they share a common DAergic deficit. Also, ADHD separately and ADHD together with RLS have been found to be associated with sleep disorders such as insomnia and a common genetic polymorphism [79-81]. In a recent study, 64% of children with ADHD were estimated to suffer from RLS judged by their nocturnal periodic limb movement [82]. It has been shown that MPH reduces total sleep time but improves sleep quality by consolidating sleep in adults [83]. However, the effect of MPH on RLS in ADHD children has never been investigated. In view of the expected inhibitory effect of MPH on DA metabolism it is important to investigate the occurrence and severity of RLS and sleep disorders in children and compare these to adults, and the effect of MPH thereupon. Sleep disorders and RLS are effective and non-invasive outcome measures to evaluate the effect of age following MPH treatment in the human brain. Therefore, we will assess RLS severity and sleep quality in the ePOD-MPH trial using questionnaires (Cambridge-Hopkins RLS questionnaire(CH-RLSq, International RLS severity scale (iRLSS), John Hopkins RLS severity scale (JH-RLS-ss), Epworth sleepiness scale (ESS) and the the Holland Sleep Diagnostic List (HSDL)) [84] and sleep log and actigraphy at three time points during the study: the week prior to the trial, during the trial, and during the washout period. Actigraphy is a non-invasive method of monitoring human rest/activity cycles. To measure gross motor activity, each patient will wear a small actigraph unit, also called an actimetry sensor, for five consecutive days. We hypothesize that due to an expected long-term reduction in DA turnover rate after early MPH treatment, there will be long lasting positive effects on RLS symptoms and sleep disorders only in children, but not adults.

#### ***Cortisol measurements***

In the ePOD-SSRI study salivary cortisol levels will be determined in salivary samples taken at home on a ‘normal’ weekday in the week before baseline and washout assessment days in order to determine the cortical awakenings response (CAR) and the diurnal cortisol cycle. Samples will be collected at 5 different moments: 1) directly after waking up, 2) 30 minutes after waking up, 3) 4 hours after waking up, 4) 8 hours after waking up, and 5) and 12 hours after waking up. To determine the peak after a 5-HT challenge, one salivary sample will be collected before the MRI scan session (baseline measure) and a second sample 30 minutes after the 5-HT challenge (directly after the MRI scan) on the day of both the MRI scan sessions.

#### ***Potential confounders***

The study is designed to limit several important possible confounding parameters, such as gender effects (only women are included in the FLX trial and only men in the MPH trial) and aging effect (small age range, only young adults included). A within subject approach (pre- and post-treatment measurement in every subject) is used to rule out most between subject differences in the RCTs. Because of the design of the study, we have limited power and can correct for a maximum of 2 or 3 confounders. Therefore, age (in months) and ratings of symptom severity will be taken into account as covariates. In addition in the ePOD-SSRI study, the 5-HTTLPR polymorphism will be determined. The long allele of this SERT polymorphism in the promoter region (5-HTTLPR) has an activity twice that of the short allele [85], resulting in higher densities of SERT. It is expected to be an important confounder to take into account when measuring SERT functioning. Also, significant associations between the long variant and a favorable treatment response have been repeatedly reported [86].

#### ***Power analysis***

Since these trials are the first to examine 5-HT and DA functioning following FLX and MPH treatment in children and young adults using MR imaging, there is only limited and indirect data available to perform a sample size calculation. The goal of our research is to detect differences in the age-dependency effect of FLX and MPH on the outgrowth of the DA-ergic and 5-HT-ergic system if these differences are in the magnitude of a standardized effect size of 1.25. From pilot experiments in rats and studies in humans with known alterations of 5-HT and/or DA (e.g., MDMA users or d-amphetamine users) we presume that the expected differences with our methods will lead to standardized effect sizes of at least 1.25. Both current trials will have the benefit of having before and after treatment measurements data from each patient. This paired data will reduce the between subject variability. This will increase the power of our trial to detect differences between groups. A sample size of 15 patients in each treatment-by-age group (4 groups) will be sufficient to detect standardized effect size of 1.25 with a two-sided significance level of 5% and a power of 90% to demonstrate age-dependency of the effects of MPH and FLX. To account for an expected drop-out of 25%, we will include 20 patients in each group for the FLX trial. Because the expected drop-out in the MPH trial is probably higher, due to motion artifacts in MRI scanning, we will include 25 patients in each treatment-by-age group. Because of slightly higher subject variability in the ePOD-Pharmo study (age and duration of treatment) again a sample size of 25 was chosen.

#### ***Statistical analysis***

**Figure 2:**
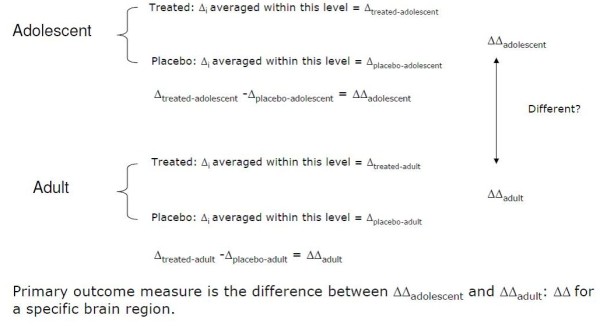
‘Comparison of mean ∆ in treated patients minus mean ∆ in placebo treated patients’.

To evaluate the age-dependency of the effect of MPH and FLX on the outgrowth of the DA-ergic and 5-HT-ergic system, the change in our primary outcome measures (CBF) from baseline to post-treatment will be determined for each patient (∆i). These individual changes (∆i) will be used to estimate the treatment effect in adolescents (mean ∆ in treated patients minus mean ∆ in placebo treated patients) and in adults, which will be compared, as shown also in Figure 2. All analysis will initially be conducted using the intension-to-treat principle, but for the imaging outcomes a per-protocol analysis will also be performed.

The central analysis examines whether this treatment effect is different in adolescents compared to adults (effect modification or interaction by age). This hypothesis will be formally examined using ANOVA. The model includes treatment group (2 categories), age group (2 categories), and the interaction between treatment and age to examine whether the impact of MPH and FLX treatment differs by age. Depending on the imaging modality we will use a whole brain voxel based analysis or an ROI analysis. The same approach can be used for explorative analysis on the age-dependency of the effects on secondary outcome measures such as behavioral outcome (fMRI, neuropsychological assessment) and behavioral measures, and cortisol response for the FLX trial and sleep-log actigraph for the MPH trial.

### Ethical considerations

Evidently, there are important ethical considerations that need to be taken into account with medication studies in children. In our case, the most important restriction is the duration of the clinical trial, or the time that a child would not receive adequate treatment (placebo condition). The duration of the RCT could not be longer than the time a child would otherwise also not receive adequate treatment, due to (relatively) long waiting lists in the Netherlands: typically 4 months at the time these studies were being evaluated by the Central Committee on Human Research in the Netherlands (CCMO). In the MPH study we overcome the treatment delay by including patients from the waiting list and offering psycho-education when necessary. In addition, in the ePOD-SSRI trial we give at least 18 sessions Cognitive Behavioral Therapy (CBT) to all adolescent participants. Therapy will be in accordance with the ‘Doepressie’ protocol, a psychotherapeutic program which is a Dutch translation of the internationally well-used program ‘Coping with Depression Course for Adolescents’ [87]. CBT is not part of standard clinical practice in the adult MDD population and will therefore not be provided to the adult patients. Adult MDD patients, who already receive some form of behavioral therapy at the start of the study, may continue this if they wish, but adult MDD patients cannot start a new therapy. Moreover, studies with SSRI’s, especially in children have shown a placebo response up to 40% making treatment with placebo more ethically acceptable.

The RCTs have been approved by the Central Committee on Human Research in the Netherlands (CCMO), the Pharmo cohort study has been approved by the local medical ethics committee (METC) of the Academic Medical Center Amsterdam (AMC). All subjects participate on a voluntary base and receive a small financial compensation (50 euro and travel expenses). Written and informed consent from both patients and legal caregivers will be obtained in all cases.

## Discussion

In the ePOD project we propose a set of neuroimaging studies and neuropsychological assessments in which we examine the neural circuitry in adolescents with depression or anxiety and ADHD before and after treatment. As pointed out recently in an editorial from the American Journal of Psychiatry [88], this type of research is greatly needed in a field in which most imaging studies have been conducted in adults. Because of ongoing brain development during adolescence, the neuropathophysiology, let alone the treatment, that underlie these disorders could be distinct. Slowly emerging evidence suggests that the long-term effects of drug exposure are delayed and expressed once the vulnerable system reaches maturation (i.e., typically during adulthood). This phenomenon, known as neuronal imprinting, occurs when the effects of drug exposure outlast the drug itself [44]. Thus, understanding the persistent effects critically depends on the window of observation. Therefore, ePOD is a unique clinical study in children and adults which will exactly grab this window of opportunity to measure age related effects of psychotropic drugs with sophisticated neuroimaging techniques. Embracing this concept should influence how we conduct preclinical assessments of developmental drug exposure, and ultimately how we conduct clinical assessments of drug efficacy, effectiveness, and safety for the treatment of childhood psychiatric disorders [12].

As the safety of antidepressants to children still is a subject of concern, particularly since FLX is now licensed for the treatment of MDD in children of 8 years and older, information about the safety of FLX in treating childhood depression is needed. Especially the potential for an increased suicide risk in association with SSRIs in general has led to much debate [89], as has also been pointed out by the Medicines Evaluation Board of the Netherlands [90] and several comments in the Lancet in response to an article by Ebmeier and colleagues [91].

The neurotransmitter 5-HT plays a crucial role in axonal outgrowth of 5-HT projections during brain development [63]. Earlier animal work demonstrated that postnatal 5-HT pharmacological manipulations can lead to abnormal outgrowth of the 5-HT system [92-94]. As an SSRI, FLX increases extracellular 5-HT concentrations by blocking SERT. Recently, studies in non-human primates have shown that FLX persistently upregulates SERT, but not 5-HT1A receptors, in the neocortex and the hippocampus of non-human primates [7]. These findings are in line with pilot experiments of our group and findings of Wegerer et al. and Bock et al., in rats, which also indicated that this effect persists into adulthood, long after discontinuation of treatment with SSRIs [5,6]. Also, we showed with phMRI that juvenile-treated rats respond more strongly to a 5-HT challenge than same-age untreated rats, while adult-treated rats show a diminished response after previous chronic treatment [48]. This study showed that the phMRI technique is very well suited to address the primary objective of the ePOD-MPH studies: investigating whether the effect(s) of FLX on serotonin depend upon age. As may be expected, on a behavioural level, results are less consistent, although age-dependent responses to SSRIs on depression-like behaviour are described in both rats and mice [2,95,96]. All these findings most likely reflect the earlier described neuronal imprinting effects.

MPH is being prescribed to increasingly younger children [97,98]. A meta-analysis has shown that in the USA and Australia up to 18 – 66% of those treated with stimulants do not meet the criteria for ADHD [99]. The increased prescription rates and concerns about proper diagnostic protocols have led to much public debate on the safety of MPH in the treatment of children. Indeed, a meta-analysis has shown that non-compliance is estimated at 20-65% and is attributed in part to apprehension about the safety of psychostimulants [100]. Recent work on the effects of MPH has shown that it may indeed normalize rates of cortical thinning, especially that of the prefrontal cortex [13]. In addition, in adult ADHD several reports on grey matter reductions were not able to distinguish between ADHD and psychostimulant effects [101,102]. However, reports on greater rates of depression and anxiety in the treated groups of the MTA study sample and in several studies involving rats indicate that effects of MPH treatment may have mixed positive and negative effects [9,10,103]. Our main outcome parameter phMRI may be able to shed more light on the effects of MPH on the development of the DAergic system. This will increase our understanding of the safety and working mechanisms of MPH in a vulnerable population. In addition, we will gain insight into basal neurocognitive and neuroadaptive processes in the developing brain, as well as increasing our knowledge on the pathophysiology of ADHD.

However, there are also some limitations of the present study designs that need to be mentioned. One limitation is that the treatment provided to adolescents and adults is not the same in the ePOD-SSRI RCT. Adolescent subjects will receive CBT, whereas adult patients will not. As mentioned previously, CBT is not part of standard clinical practice in the adult MDD population and will therefore not be provided to the adult patients. From a methodological point of view it would have been ideal to isolate the effect of FLX and add no other treatment than this one in both age groups. However, this is not ethical as CBT is always part of standard clinical practice in the Netherlands in adolescent patients suffering from MDD. However, since change scores from baseline to post-treatment will be determined for each patient (∆i), the potential effect of (the lack) of CBT will be minimized. Another limitation is that no conclusions from the ePOD-SSRI and ePOD-MPH RCTs can be made on the long-term effects of these medications on brain development. The RCTs last for ‘only’ 4 months, and the washout period is 3 weeks maximum. For that reason, we designed the ePOD-Pharmo study, in which subjects are screened at least 7 years later following early FLX or MPH exposure. In addition, all participants in the RCTs are asked if they are willing to participate in a follow up study, scheduled in 3–5 years, and most are willing to participate. Thus, by combining the RCTs in which we investigate the *causality* of the age-dependency of FLX and MPH, together with the ePOD-Pharmo study which is directed towards the *long-term effects* of these medicines, will ultimately provide missing knowledge.

As recently indicated by Tao and colleagues, studies are needed that use the same methodology simultaneously in both adolescents and adults, to overcome methodological differences, and correct interpretation of the age-dependency of results [104]. Sample differences in age and illness status or differences in the image acquisition/analysis approach may obscure the age-dependency of the findings. These issues are overcome by the current study design. Since this study employs randomized controlled trials and has the benefit of having before and after treatment measurements from each patient, we will be able to reduce subject variability. This increases the ability of our trial to detect differences between groups. Moreover, this study employs novel non-invasive MRI techniques in children and adolescents, which provide new insights into the effects of psychotropic drugs on the developing brain. The use of phMRI in assessing DAergic and 5-HTergic functionality may have important prognostic factors, for instance in predicting responsiveness to psychostimulants or antidepressant medication in the near future.

## Conclusion

So far, most imaging studies have been conducted in adults. Ongoing brain development during adolescence may distinct the neural mechanisms that underlie psychiatric disorders like depression, anxiety and ADHD. Examination of these mechanisms during early phases of the disorder provides the opportunity to avoid confounds due to complex treatment histories or potential scarring from years of disease. A better understanding of adolescent-specific mechanisms will be “a critical foundation for the advancement of early treatment interventions, which could significantly affect public health” [88].

In the ePOD studies we propose a set of neuroimaging studies and neuropsychological assessments in which we examine the neural circuitry in adolescents with depression or anxiety and adolescents with ADHD before and after treatment. The combination of prospective studies with a cross-sectional cohort study, using the same outcome measures, will increase our understanding not only of the working mechanisms of both FLX and MPH in children and adolescents, but also provide more information about the safety of these substances in the maturing brain.

## Competing interests

The authors declare that they have no competing interests.

## Authors’ contributions

Conception of the study was conducted by LR. All authors participated in design of the study. MB, MS, AK, HT, AS, and CB acquire the data. MB, MS, AK, HT, AS, CB, MdR and HR will analyse the data. MB, MS, AK, HT, AS, CB, MdR, RR, HR, HG and LR will participate in data interpretation. MB, MS, AK and LR drafted the manuscript. All authors revised the manuscript critically for important intellectual content. All authors read and approved the final manuscript.

## Pre-publication history

The pre-publication history for this paper can be accessed here:

http://www.biomedcentral.com/1471-244X/14/48/prepub
